# Decision-Making of Irrigation Scheme for Soybeans in the Huaibei Plain Based on Grey Entropy Weight and Grey Relation–Projection Pursuit

**DOI:** 10.3390/e21090877

**Published:** 2019-09-09

**Authors:** Yi Cui, Shangming Jiang, Juliang Jin, Ping Feng, Shaowei Ning

**Affiliations:** 1 Stage Key Laboratory of Hydraulic Engineering Simulation and Safety, Tianjin University, Tianjin 300072, China (Y.C.) (P.F.); 2 Key Laboratory of Water Conservancy and Water Resources of Anhui Province, Water Resources Research Institute of Anhui Province and Huaihe River Commission, Ministry of Water Resources, Hefei 230088, China; 3 School of Civil Engineering, Hefei University of Technology, Hefei 230009, China

**Keywords:** irrigation scheme decision-making, system comprehensive evaluation, grey relation analysis, entropy weight, projection pursuit, soybean, potted experiment, Huaibei Plain

## Abstract

To provide a scientific reference for formulating an effective soybean irrigation schedule in the Huaibei Plain, potted water deficit experiments with nine alternative irrigation schemes during the 2015 and 2016 seasons were conducted. An irrigation scheme decision-making index system was established from the aspects of crop water consumption, crop growth process and crop water use efficiency. Moreover, a grey entropy weight method and a grey relation–projection pursuit model were proposed to calculate the weight of each decision-making index. Then, nine alternative schemes were sorted according to the comprehensive grey relation degree of each scheme in the two seasons. The results showed that, when using the entropy weight method or projection pursuit model to determine index weight, it was more direct and effective to obtain the corresponding entropy value or projection eigenvalue according to the sequence of the actual study object. The decision-making results from the perspective of actual soybean growth responses at each stage for various irrigation schemes were mostly consistent in 2015 and 2016. Specifically, for an integrated target of lower water consumption and stable biomass yields, the scheme with moderate-deficit irrigation at the soybean branching stage or seedling stage and adequate irrigation at the flowering-podding and seed filling stages is relatively optimal.

## 1. Introduction

Soybeans (*Glycine max* (L.) Merrill) are an important food and oil crop [1] and also a substantial source of high-quality protein for humans [2]. Meanwhile, with the development of social economy and the improvement of living standard, the demand for soybeans has been rapidly increasing. Huaibei Plain is a main planting region for high-protein soybeans in China, the average annual planting area is 0.7–0.8 million hectares [3]. However, the Huaibei Plain is located in a typical monsoon climate zone and has a non-uniform temporal distribution of precipitation, the water requirement during the soybean growth period should be mostly supplemented by irrigation for stable yields. The average water resources amount per capita in this region is 530 m^3^, which is one fourth that in China [4]. Moreover, it is likely that a severe water shortage period will happen here in the future due to global climate change and local drying tendencies [5,6]. Therefore, identifying the quantitative responses of soybean growth to water deficit at each stage and proposing a relatively optimal irrigation scheme are fundamental to formulate an accurate irrigation schedule and improve the productivity of limited water resources for soybeans in the Huaibei Plain.

Regulated deficit irrigation is an irrigation mode proposed by the Australian Institute of Sustainable Irrigated Agriculture in the mid-1970s [7,8]. This mode aims at reducing water consumption meanwhile guaranteeing grain yields by regulating plant growth during the vegetative phase and reallocating the proportions of photosynthetic organic matter between vegetative and reproductive organs [9,10]. At present, the studies on the influences of water deficit on soybean growth, development and yield formation by setting various deficit irrigation treatments in field experiments have been widely conducted [11,12,13,14]. However, these studies mostly focus on the quantitative responses of soybean evapotranspiration, biomass, seed yield or water use efficiency to different intensity levels and occurrence periods of water deficit, and do not further propose a reasonable irrigation scheme by integrating these responses. In addition, suitable soybeans regulated deficit irrigation or supplemental irrigation schemes have been gradually presented and evaluated based on field experiments and modeling simulations [15,16,17,18]. Nevertheless, most of the schemes are determined merely from a single index aspect, such as soybean yield or production benefit, the index that depicts the effect on plant growth process has rarely been considered. There is a lack of a systematic and scientific decision-making multi-index system that considers the integrated influence of an irrigation scheme on crop water consumption, crop growth process and crop water use efficiency. Therefore, it is necessary to establish a relatively complete decision-making index system combined with the water deficit experiments, which could precisely reflect the actual soybean growth responses at each stage, for various irrigation schemes.

Irrigation scheme decision-making is to select a relatively optimal irrigation scheme from lots of scheme samples, which could be regarded as a system evaluation problem [19]. Grey relation analysis is an effective scheme decision-making method, it quantifies the grey relation degree between an alternative scheme and the ideal scheme by considering the similarity or difference degree of each decision-making index between the two schemes. This method has been widely used to solve decision-making issues in materials science [20], computer science [21], management science [22] and environmental science [23] fields. However, grey relation analysis has not been applied to soybean irrigation scheme decision-making in the Huaibei Plain. Moreover, for the grey relation analysis model that consists of multiple decision-making indices, the weight of each index should be reasonably determined. Nevertheless, there is a lack of a general method with strong applicability for calculating index weight in comprehensive decision-making problems. In recent years, information entropy theory has been used to obtain index weight [24,25,26]. However, the proportion of each index in the original research issue is often changed when applying this method [27,28]. Furthermore, almost all of the entropy weight studies directly use the sequence of original index values to calculate the corresponding entropy value. However, for some problems, the study object is not the original index, but a variable converted from the original index; if the sequence of original index values is used to obtain the entropy value, it may bring redundant or inaccurate information and reduce the validity of the entropy weight method. Similarly, for the projection pursuit model, which is also a powerful means to determine index weight [29,30], most of the relevant studies directly use the sequence of original index values to construct the projection eigenvalue. It may be not in accordance with the projection regulation for a specific research problem. Therefore, it is crucial to identify and incorporate the actual study object when using the entropy weight method or projection pursuit model for calculating index weight. In this study, actual water deficit experiments in pots with various alternative irrigation schemes during two cropping seasons were implemented to (1) establish a relatively complete decision-making index system by considering the integrated influence of an irrigation scheme on crop water consumption, crop growth process and crop water use efficiency; (2) build object-oriented entropy weight and projection pursuit models to respectively determine the weight of each decision-making index based on the corresponding grey relation coefficient sequence obtained by grey relation analysis theory; and (3) propose a relatively optimal irrigation scheme according to the comprehensive grey relation degree of each alternative scheme for supporting an effective and accurate soybean irrigation schedule in the Huaibei Plain.

## 2. Materials and Methods

### 2.1. Experimental Site

**Figure 1:**
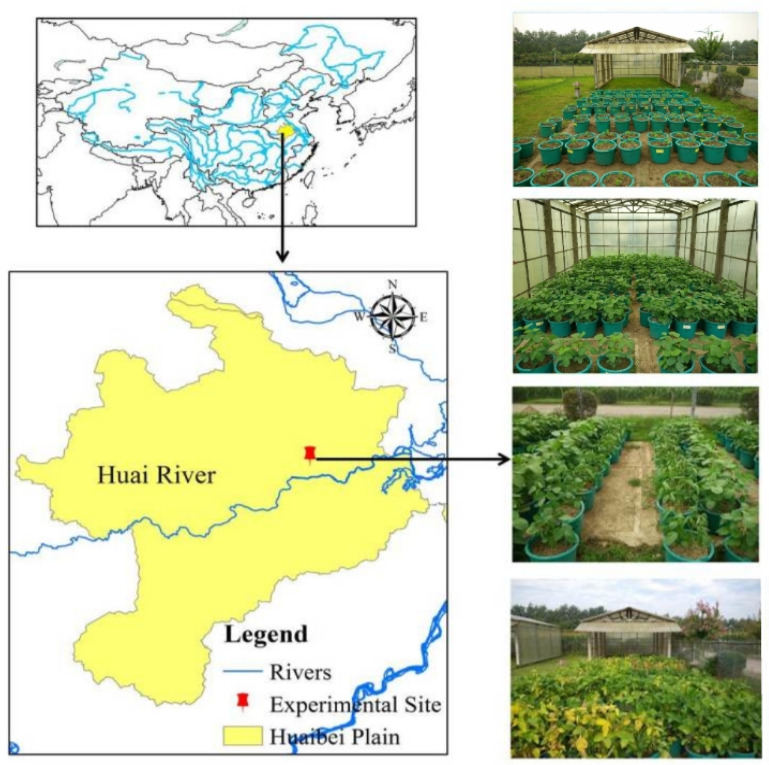
Location of the site for conducting soybean potted deficit irrigation experiments.

**Figure 2:**
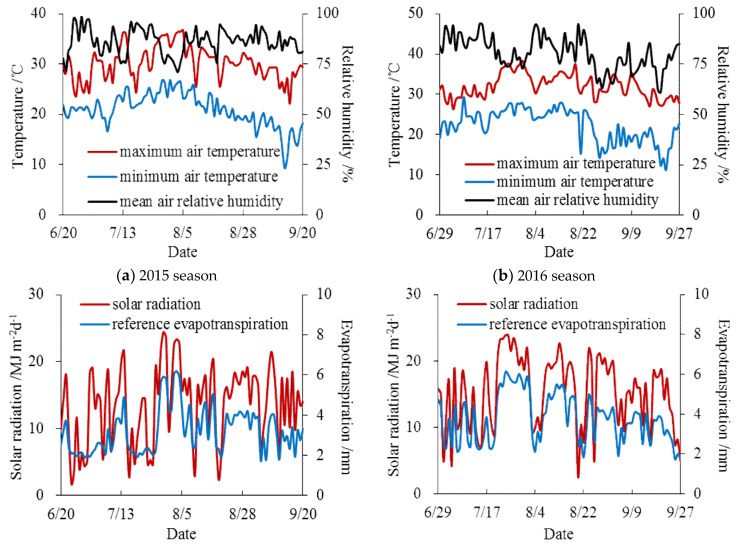
Daily climatic conditions during the whole growth periods of soybean in (**a**) 2015; (**b**) 2016.

Potted water deficit experiments were conducted in the Xinmaqiao Agricultural Irrigation Research Station, Water Resources Research Institute of Anhui Province and Huai River Commission, P.R. China (Figure 1). This region has a typical northern subtropical and warm, temperate transition zone climate [3], the soybeans are planted in summer and almost under rainfed conditions by local farmers [31], which frequently causes great production losses from drought disaster due to an uneven temporal precipitation distribution. The soybean deficit irrigation experiments were both implemented from June to September in 2015 and 2016, and the basic climatic conditions during the whole growth periods of soybean in the two seasons are shown in Figure 2.

### 2.2. Crop Management

Soybeans were planted in pots with an upper diameter of 28 cm, a bottom diameter of 20 cm and a height of 27 cm, with 15 kg of air-dried soil loaded into each empty pot in 2015. Nevertheless, for the 2016 season, the upper diameter, bottom diameter and height of the pots became 31 cm, 23 cm and 27 cm, respectively, and, meanwhile, the amount of air-dried soil increased to 17 kg. All empty pots were weighed before adding the air-dried soil. For both seasons, the experimental soil was collected from a field tillage layer at this site and was a typical Shajiang black soil in the Huaibei Plain. The characteristics of this soil at the upper layer (0–50 cm) [3] are shown in Table 1.

During the 2015 and 2016 seasons, the cultivars of soybean seeds for experiments were both Zhonghuang-13. This was a high-protein soybean cultivar bred by the Institute of Crop Sciences, Chinese Academic of Agricultural Sciences, and was widely planted in the Huang-Huai-Hai region, China [32]. The cultivar parameters of this soybean seed [32,33] are shown in Table 2.

In 2015, seeds were sown on June 20 and did not all germinate until July 3. Then, experimental treatments were implemented from July 4 to September 20 (harvest date). During the 2016 season, the same cultivar seeds were sown on June 29 and did not all germinate until July 14, treatments were conducted from July 15 to September 27. According to the soybean planting density in field around the Huaibei Plain, each pot retained three plants. Combining years of actual soybean growth records in this site with relevant studies on soybean growth stages [13,34], the whole soybean growth period from sowing to harvest was divided into five single stages for both seasons (Table 3).

To ensure the germination of seeds, the soil water content in each soybean pot was irrigated to field capacity after sowing along with 4 g of compound fertilizer (N 15%, P_2_O_5_ 15%, K_2_O 15%). All pots were placed in an open environment under a movable shed that was closed when precipitation occurred (Figure 1). During the experimental period, except for irrigation, other crop management practices were the same for all soybean pot samples. Moreover, the mean daily meteorological elements during each soybean growth stage for both seasons are shown in Table 4.

### 2.3. Irrigation Scheme Design

For both seasons, there were one full irrigation scheme (CK) and eight deficit irrigation schemes (T1–T8) in the experiments (Table 5). To implement irrigation scheme decision-making based on the quantitative responses of soybean growth process to drought stress during different periods, deficit irrigation at each single stage (after seed germination) was conducted. Moreover, alternative schemes were designed by setting different lower limits of soil water content in the pots at four stages. According to years of crop deficit irrigation experiments in this site and the previous studies [12,34], three lower limits of soil water content (35%, 55% and 75% of field capacity) were set, which corresponded to serious water deficit, slight water deficit and no water deficit treatments, respectively. In addition, to be close to the actual irrigation mode in production, soybean plants were irrigated to 90% of field capacity once the soil water dropped below the lower limit [35]. Specifically, slight and serious water deficit treatments were, respectively, set up at the seedling stage, the branching stage, the flowering-podding stage and the seed filling stage and were referred to as deficit irrigation schemes T1–T8. No water deficit treatment was set up during the whole growth period of soybean, which was referred to as full irrigation scheme CK in 2015 and 2016 (Table 5).

Except for the soybean pots used to measure yield components at harvest (fifteen and five replications for CK during the 2015 and 2016 seasons, five replications for T1–T8 in the two seasons), five additional replications were arranged for measuring plant biomass at the end of the seedling stage, the branching stage and the flowering-podding stage, respectively. Furthermore, considering the influence of soybean growth on calculating soil water content, the plant weight at the end of the previous stage should be subtracted from the pot weight during the current stage. Soybean pots were both arranged in a completely randomized experimental design for the two seasons (Figure 1).

### 2.4. Measurements

(1). Pot weight

*W_j_* is the weight of a soybean pot on day *j* after seed germination (kg), which was measured by an electronic balance. All pots were weighed at 6 pm from seed germination to plant harvest.

(2). Soil water content

The soil water content in each pot was calculated according to the pot weight as follows:

(1) $$\theta_{j,b}=\frac{W_{j-1}-W_{s}-W_{p}+I_{j}}{W_{s}},$$

(2) $$\theta_{j,e}=\frac{W_{j}-W_{s}-W_{p}}{W_{s}},$$

(3) $$\theta_{j}=\frac{\theta_{j,b}+\theta_{j,e}}{2},$$

where *θ_j_*_,b_ is the soil water content in a soybean pot at the beginning of day *j*, immediately after irrigation (g g^−1^ of soil dry weight); *θ_j_*_,e_ is the soil water content at the end of day *j* when weighing the pot (g g^−1^ of soil dry weight); *θ_j_* is the average soil water content on day *j* (g g^−1^ of soil dry weight); *W*_p_ is the weight of the empty pot (kg); *W*_s_ is the weight of air-dried soil that was loaded into the pot (kg); and *I_j_* is the irrigation amount for the pot on day *j* (kg).

(3). Irrigation amount

Whether the soybeans needed to be irrigated was determined by comparing the soil water content in a pot and the corresponding lower limit. The irrigation amount was calculated as follows:

(4) $$I_{j}=\{\begin{array}{c} 0\;\;\;\;\;\;\;\;\;\;\;\;\;\;\;\;\;\;\;\;\;\;\;\;\;\;\;\;\;\;\theta_{j-1,e}\geq \theta_{\mathrm{lm}}\\ (90\%\theta_{\mathrm{FC}}-\theta_{j}{}_{-1,e\;})\times W_{s}\;\;\;\;\theta_{j-1,e}<\theta_{\mathrm{lm}} \end{array},$$

where *θ*_FC_ is the soil water at field capacity (g g^−1^ of soil dry weight); *θ_j_*_−1,e_ is the soil water content in a pot at the end of day (*j* − 1) (g g^−1^ of soil dry weight); and *θ*_lm_ is the corresponding lower limit of soil water content for experimental treatment of the pot (g g^−1^ of soil dry weight). The irrigation amount was metered by measurement and implemented at 7 am.

(4). Soybean water consumption

The actual evapotranspiration of soybean in each pot was calculated according to the pot weight and irrigation amount by the following formula:

(5) $$ET_{c,j}=W_{j-1}+I_{j}-W_{j},$$

where *ET_c_*_,*j*_ is the evapotranspiration of soybean on day *j* (mm)—it could be converted from kg.

(5). Aboveground biomass and seed yield

Soybean aboveground biomass of three plants in a pot were measured at the end of each growth stage (after seed germination) by breaking the pot. The aboveground accumulated biomass at a given stage was the difference of biomass between this stage and the previous stage. Seed yield and number of seeds in a pot were measured at harvest, and 1000 seed weight was obtained. Seed yield and aboveground biomass were measured by an electronic balance after drying in the sun.

### 2.5. Irrigation Scheme Decision-Making Model

**Figure 3:**
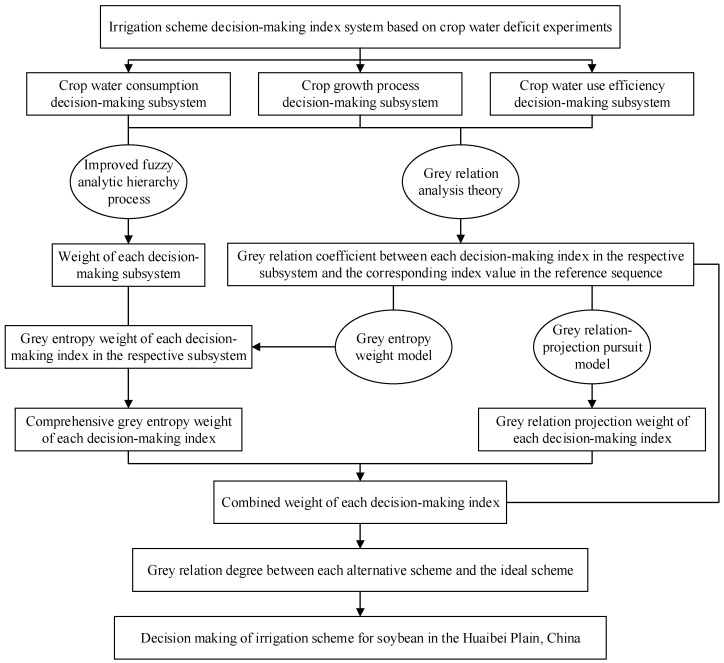
Process of soybean irrigation scheme decision-making in the Huaibei Plain.

The process to establish the soybean irrigation scheme decision-making model in this study included the following seven steps (Figure 3):

Step 1: According to the targets of decision-making, the irrigation scheme decision-making index system was divided into three aspects of crop water consumption, crop growth process, and crop water use efficiency. Specifically, the index system could be denoted as {x^*^(*k*, *j*)|*k* = 1, 2, 3; *j* = 1, 2, …, *n_k_*}, where x^*^(*k*, *j*) was the decision-making index *j* in the *k*-th decision-making subsystem; *n_k_* was the number of indices in the *k*-th subsystem; *k* = 1, 2, 3, respectively, represented crop water consumption, crop growth process, and crop water use efficiency subsystems; and *n* was the total number of decision-making indices and *n* = *n*_1_ + *n*_2_ + *n*_3_. Therefore, the samples of irrigation scheme decision-making index were described as {x^*^(*i*, *k*, *j*)|*i* = 1, 2, …, *N*; *k* = 1, 2, 3; *j* = 1, 2, …, *n_k_*}, where *N* was the number of alternative irrigation schemes. The normalized samples *x*(*i*, *k*, *j*) were obtained according to Equations (6) and (7).

For an index (positive index), the larger the index value was, the more efficient the irrigation scheme was. This index value was normalized by the following formula [29]:

(6) $$x\left(i,k,j\right)=\frac{x^{*}\left(i,k,j\right)-\underset{i}{\min}x^{*}(i,k,j)}{\underset{i}{\max}x^{*}(i,k,j)-\underset{i}{\min}x^{*}(i,k,j)},$$

where $\underset{i}{\min}$ x^*^(*i*, *k*, *j*) and $\underset{i}{\max}$ x^*^(*i*, *k*, *j*) represent the minimum and maximum values of the decision-making index *j* in the *k*-th subsystem among all irrigation schemes, respectively.

Additionally, for an index (negative index), the smaller the index value was, the more efficient the scheme was. This index value was normalized as follows [29]:

(7) $$x\left(i,k,j\right)=\frac{\underset{i}{\max}x^{*}(i,k,j)-x^{*}\left(i,k,j\right)}{\underset{i}{\max}x^{*}(i,k,j)-\underset{i}{\min}x^{*}(i,k,j)}.$$

Step 2: Grey relation analysis (GRA) as proposed by Deng [36], is an effective scheme decision-making method. First, the reference sequence of the ideal irrigation scheme {*x*_0_(*k*, *j*) = $\underset{i}{\max}$*x*(*i*, *k*, *j*)|*i* = 1, 2, …, *N*; *k* = 1, 2, 3; *j* = 1, 2, …, *n_k_*} was generated, by taking the largest normalized value of each decision-making index in the respective subsystem among all alternative irrigation schemes. Then, the absolute difference between a sample sequence and the reference sequence was obtained as follows [20]:

(8) $$\Delta \left(i,k,j\right)=\left|x_{0}\left(k,j\right)-x\left(i,k,j\right)\right|,$$

where ∆(*i*, *k*, *j*) is the absolute difference between the index *j* in the *k*-th subsystem for the scheme *i* and the corresponding index value in the reference sequence.

Accordingly, the grey relation coefficient between the index *j* in the *k*-th subsystem for the scheme *i* and the corresponding index value in the reference sequence *ξ*(*i*, *k*, *j*) was determined as follows [20]:

(9) $$\xi \left(i,k,j\right)=\frac{\underset{i}{\min}\;\underset{j}{\min}\Delta \left(i,k,j\right)+\lambda \underset{i}{\max}\;\underset{j}{\max}\Delta \left(i,k,j\right)}{\Delta \left(i,k,j\right)+\lambda \underset{i}{\max}\;\underset{j}{\max}\Delta \left(i,k,j\right)},$$

where $\underset{i}{\min}$ $\underset{j}{\min}$ ∆(*i*, *k*, *j*) and $\underset{i}{\max}$ $\underset{j}{\max}$ ∆(*i*, *k*, *j*) represent the minimum and maximum absolute differences among all indices in the *k*-th subsystem for all schemes, respectively; *λ* is the distinguishing coefficient, which is selected from 0 to 1. In this study, *λ* took 0.5 for guaranteeing a good calculation stability and a moderate distinguishing ability [20,36].

Step 3: An improved fuzzy analytic hierarchy process based on the accelerating genetic algorithm (AGA-FAHP) [28] was used to calculate the weight of each subsystem {*w*_sub,*k*_|*k* = 1, 2, 3}.

The experts were invited to compare the importance of crop water consumption, crop growth process, and crop water use efficiency decision-making subsystems in pairs, and, then, the fuzzy complementary judgment matrix *A*_sub_ = (*a_kl_*)_3×3_ was obtained. The AGA-FAHP method was used to test and correct the consistency of *A*_sub_ and calculate *w*_sub,*k*_. Specifically, if *A*_sub_ satisfied the full consistency, the following equation would be established [28]:

(10) $$\sum_{k=1}^{3}\sum_{l=1}^{3}\frac{\left|0.5\left(3-1\right)\left(w_{\mathrm{sub},k}-w_{\mathrm{sub},l}\right)+0.5-a_{kl}\right|}{3^{2}}=0,$$

where the left item in Equation (10) is the consistency index of *A*_sub_. If the result of this consistency index was less than a critical value, it showed that *A*_sub_ had a satisfactory consistency; otherwise, *A*_sub_ should be corrected. The corrected *A*_sub_ was denoted as *B*_sub_ = (*b_kl_*)_3×3_*,* and the ordering weights of element in *B*_sub_ were still recorded as {*w*_sub,*k*_|*k* = 1, 2, 3}. Furthermore, *B*_sub_ met the following formula [28]:

(11) $$\min CIC\left(3\right)=\sum_{k=1}^{3}\sum_{l=1}^{3}\frac{\left|b_{kl}-a_{kl}\right|}{3^{2}}+\sum_{k=1}^{3}\sum_{l=1}^{3}\frac{\left|0.5\left(3-1\right)\left(w_{\mathrm{sub},k}-w_{\mathrm{sub},l}\right)+0.5-b_{kl}\right|}{3^{2}}\;s.t.\;\;\;\;\;\;\;\;\;\;\left\{ \begin{array}{l} b_{kk}=0.5,\;\;\;\;\;\;\;\;\;\;k=1,2,3\\ 1-b_{lk}=b_{kl}\in [a_{kl}-d,a_{kl}+d]\cap [0,1],\;\;\;\;\;\;\;\;\;\;k,l=1,2,3\\ \sum_{k=1}^{3}w_{\mathrm{sub},k}=1.0,w_{\mathrm{sub},k}\in [0,1],\;\;\;\;\;\;\;\;\;\;k=1,2,3 \end{array}\right.,$$

where *B*_sub_ is regarded as the optimal fuzzy consistency judgment matrix of *A*_sub_ when the result of *CIC* reached a minimum value; *CIC*(3) is the consistency index coefficient; *d* is a non-negative parameter and selected from 0 to 0.5 for guaranteeing the relationship of importance between two decision-making subsystems [28].

When the result of *CIC*(3) was less than a critical value, it indicated that *A*_sub_ had a satisfactory consistency and the obtained subsystem weights were acceptable; otherwise, the parameter *d* was adjusted until *A*_sub_ met a satisfactory consistency. Based on plenty of numerical experiments and relevant research [28,37], the matrix was considered to have a satisfactory consistency when the value of *CIC* was less than 0.20 in this study.

Step 4: A grey entropy weight method combined with the AGA-FAHP was proposed to determine the weight of each index in the respective subsystem {*w*_e_(*k*, *j*)|*k* = 1, 2, 3; *j* = 1, 2, …, *n_k_*}.

The grey relation coefficient between the index *j* in the *k*-th subsystem for the scheme *i* and the corresponding index value in the reference sequence, could be converted into a probability variable *p*(*i*, *k*, *j*) based on information entropy theory as follows:

(12) $$p\left(i,k,j\right)=\frac{\xi \left(i,k,j\right)}{\sum_{i=1}^{N}\xi \left(i,k,j\right)}.$$

Then, the corresponding entropy value *e*(*k*, *j*) was obtained by the following formula [38]:

(13) $$e\left(k,j\right)=-\frac{\sum_{i=1}^{N}p\left(i,k,j\right)\ln p\left(i,k,j\right)}{\ln N}.$$

Considering the consistency among the initial weight of each index reflected by entropy value, a complementary judgment matrix *A*_e_*^k^* = (*u_jq_^k^*)*_n__k_*_×*n*_*_k_* was built as follows:

$$A_{e}^{k}={\left(u_{jq}^{k}\right)}_{n_{k}\times n_{k}}=\left[\begin{array}{cccc} u_{11}^{k} & u_{12}^{k} & \cdots & u_{1n_{k}}^{k}\\ u_{21}^{k} & u_{22}^{k} & \cdots & u_{2n_{k}}^{k}\\ \vdots & \vdots & \cdots & \vdots \\ u_{n_{k}1}^{k} & u_{n_{k}2}^{k} & \cdots & u_{n_{k}n_{k}}^{k} \end{array}\right].$$

Furthermore, the elements in *A*_e_*^k^* were obtained according to the following formula [28]:

(14) $$u_{jq}^{k}=\frac{1-e\left(k,j\right)}{1-e\left(k,j\right)+1-e\left(k,q\right)},\;\;\;\left(k=1,2,3;j,q=1,2,\ldots ,n_{k}\right).$$

Similarly, according to the method for determining the weight of the decision-making subsystem, the AGA-FAHP method was used to obtain the optimal consistency judgment matrix *B*_e_*^k^* = (*v_jq_^k^*)*_n__k_*_×*n*_*_k_* and the grey entropy weight of each index in the respective subsystem *w*_e_(*k*, *j*) by solving the following optimization issue [28]:

(15) $$\min CIC\left(n_{k}\right)=\sum_{j=1}^{n_{k}}\sum_{q=1}^{n_{k}}\frac{\left|v_{jq}^{k}-u_{jq}^{k}\right|}{n_{k}^{2}}+\sum_{j=1}^{n_{k}}\sum_{q=1}^{n_{k}}\frac{\left|0.5\left(n_{k}-1\right)\left[w_{e}\left(k,j\right)-w_{e}\left(k,q\right)\right]+0.5-v_{jq}^{k}\right|}{n_{k}^{2}}\;s.t.\;\;\;\;\;\;\;\;\;\;\left\{ \begin{array}{l} v_{jj}^{k}=0.5,\;\;\;k=1,2,3;j=1,2,\ldots ,n_{k}\\ 1-v_{qj}^{k}=v_{jq}^{k}\in \left[u_{jq}^{k}-d,u_{jq}^{k}+d\right]\cap \left[0,1\right],\;\;\;k=1,2,3;j\;=1,2,\ldots ,n_{k};q=j+1,j+2,\ldots ,n_{k}\\ \sum_{j=1}^{n_{k}}w_{e}\left(k,j\right)=1.0,w_{e}\left(k,j\right)\in \left[0,1\right],\;\;\;\;k=1,2,3;j=1,2,\ldots ,n_{k} \end{array}\right..$$

Moreover, the comprehensive grey entropy weight of each decision-making index {*w*_E_(*k*, *j*)|*k* = 1, 2, 3; *j* = 1, 2, …, *n_k_*} was calculated by the following formula:

(16) $$w_{E}(k,j)=w_{e}(k,j)w_{\mathrm{sub},k},\;\;\;\;\;\;\;\;\;\;k=1,2,3;j=1,2,\ldots ,n_{k}.$$

Step 5: In addition, a grey relation–projection pursuit model was also built to determine the weight of each decision-making index {*w*_P_(*k*, *j*)|*k* = 1, 2, 3; *j* = 1, 2, …, *n_k_*}. In this study, the grey relation coefficient between the index *j* in the *k*-th subsystem for the scheme *i* and the corresponding index value in the reference sequence *ξ*(*i*, *k*, *j*), was used to construct the projection eigenvalue of the scheme *i*. Specifically, a one-dimensional projection eigenvalue was obtained by synthesizing the high-dimensional data {*ξ*(*i*, *k*, *j*)|*i* = 1, 2, …, *N*; *k* = 1, 2, 3; *j* = 1, 2, …, *n_k_*} according to the grey relation–projection pursuit model as follows:

(17) $$Z\left(i\right)=\sum_{k=1}^{3}\sum_{j=1}^{n_{k}}y\left(k,j\right)\xi \left(i,k,j\right),$$

where *Z*(*i*) is the one-dimensional projection eigenvalue of an *n*-dimensional grey relation coefficient *ξ*(*i*, *k*, *j*) for the alternative irrigation scheme *i*; ***y*** = (*y*(1, 1), …, *y*(1, *n*_1_), *y*(2, 1), …, *y*(2, *n*_2_), *y*(3, 1), …, *y*(3, *n*_3_)) is the *n*-dimensional unit projection vector.

Furthermore, according to projection pursuit theory, the obtained projection eigenvalue point *Z*(*i*) should satisfy a certain distribution characteristic [29,30,39]. In detail, the distribution of local projection points within a given distance should be as concentrated as possible, and, meanwhile, the overall distribution of all projection points should be as scattered as possible. Therefore, for calculating a relatively optimal unit projection vector ***y***, the following projection index function *Q*(***y***) was established [29,30,39]:

(18) $$Q\left(y\right)=S_{Z}D_{Z},$$

where *S*_Z_ is the standard deviation of projection eigenvalue series *Z*(*i*) and *D*_Z_ is the local density of *Z*(*i*). The corresponding calculation formulas are shown as follows [29,39]:

(19) $$S_{Z}={\left[\frac{\sum_{i=1}^{N}{\left(Z\left(i\right)-\bar{Z}\right)}^{2}}{N-1}\right]}^{0.5},$$

(20) $$D_{Z}=\sum_{i=1}^{N}\sum_{m=1}^{N}\left[\left(R-r\left(i,m\right)\right)U\left(R-r\left(i,m\right)\right)\right],\;U\left(R-r\left(i,m\right)\right)=\{\begin{array}{c} 1,\;\;\;R\geq r\left(i,m\right)\\ 0,\;\;\;R<r\left(i,m\right) \end{array}\;\;,\;r\left(i,m\right)=\left|Z\left(i\right)-Z\left(m\right)\right|,$$

where $\bar{Z}$ is the average value of *Z*(*i*); *R* is the window breadth of local density and the value usually is *θS*_Z_. In this study, the value of *θ* was 0.1 [39]. *r*(*i*, *m*) represents the distance between any two projection eigenvalues *Z*(*i*) and *Z*(*m*); *U* is the unit step function, the function value is 1 when [*R* − *r*(*i*, *m*)] ≥ 0 and is 0 when [*R* − *r*(*i*, *m*)] < 0.

When the value of *Q*(***y***) reached a relative maximum, an optimal ***y*** was obtained. The question could be solved by the following optimization function based on the AGA [29,39]:

(21) $$\max Q\left(y\right)=S_{Z}D_{Z}\;s.t.\;\;\;\;\;\;\;\;\;\;\left\{ \begin{array}{l} y\left(k,j\right)\in \left[0,1\right],\;\;\;k=1,2,3;j\;=1,2,\ldots ,n_{k}\\ \sum_{k=1}^{3}\sum_{j=1}^{n_{k}}y^{2}\left(k,j\right)=1.0 \end{array}\right..$$

Correspondingly, the grey relation projection weight of each decision-making index was calculated according to the optimized ***y*** [29,30,39]:

(22) $$w_{P}(k,j)=y^{2}\left(k,j\right),\;\;\;\;\;\;\;\;\;\;k=1,2,3;j=1,2,\ldots ,n_{k}.$$

Step 6: The combined weight {*w*_C_ (*k*, *j*)|*k* = 1, 2, 3; *j* = 1, 2, …, *n_k_*} of grey entropy weight *w*_E_(*k*, *j*) and grey relation projection weight *w*_P_(*k*, *j*) for each decision-making index in the respective subsystem was obtained according to minimum relative entropy theory [40]:

(23) $$\min F=\sum_{k=1}^{3}\sum_{j=1}^{n_{k}}w_{C}\left(k,j\right)\left[\ln w_{C}\left(k,j\right)-\ln w_{E}\left(k,j\right)\right]+\sum_{k=1}^{3}\sum_{j=1}^{n_{k}}w_{C}\left(k,j\right)\left[\ln w_{C}\left(k,j\right)-\ln w_{P}\left(k,j\right)\right]\;s.t.\;\;\;\;\;\;\;\;\;\;\sum_{k=1}^{3}\sum_{j=1}^{n_{k}}w_{C}\left(k,j\right)=1,w_{C}\left(k,j\right)\in \left[0,1\right],\;\;\;k=1,2,3;j=1,2,\ldots ,n_{k}.$$

The optimization problem in Equation (23) could be further converted to the following equation according to the Lagrange multiplier method [28]:

(24) $$w_{C}\left(k,j\right)=\frac{{\left[w_{E}\left(k,j\right)w_{P}\left(k,j\right)\right]}^{0.5}}{\sum_{k=1}^{3}\sum_{j=1}^{n_{k}}{\left[w_{E}\left(k,j\right)w_{P}\left(k,j\right)\right]}^{0.5}},\;\;\;k=1,2,3;j=1,2,\ldots ,n_{k}.$$

Step 7: Finally, the grey relation degree between each alternative irrigation scheme and the ideal scheme was calculated. Furthermore, the larger the value of the grey relation degree was, the more effective the alternative scheme was. The grey relation degree for an alternative irrigation scheme was obtained by summing the product of the grey relation coefficient and combined weight for each decision-making index as follows [20]:

(25) $$G\left(i\right)=\sum_{k=1}^{3}\sum_{j=1}^{n_{k}}w_{C}\left(k,j\right)\xi \left(i,k,j\right),$$

where *G*(*i*) represents the grey relation degree between the alternative irrigation scheme *i* and the ideal scheme.

## 3. Results and Discussion

### 3.1. Irrigation Scheme Decision-Making Index Values

Based on systematic analysis of an irrigation scheme decision-making process, the actual water resources and soybean production conditions in the Huaibei Plain and relevant studies [3,4,41], a decision-making index system consisting of three subsystems (crop water consumption, crop growth process and crop water use efficiency) and sixteen decision-making indices (*X*_1_–*X*_16_) was constructed (Table 6). Meanwhile, according to the observed results of each index from the practical soybean deficit irrigation experiments in 2015 and 2016, the index samples were obtained.

Furthermore, the value of each decision-making index in Table 6 was normalized according to Equation (6) or (7) (Table 7). The reference sequence of the ideal irrigation scheme *x*_0_ = (1.00, 1.00, 1.00, 1.00, 1.00, 1.00, 1.00, 1.00, 1.00, 1.00, 1.00, 1.00, 1.00, 1.00, 1.00, 1.00), which consisted of the maximum normalized value of each index among all alternative irrigation schemes.

### 3.2. Grey Relation Coefficient of Each Decision-Making Index

**Figure 4:**
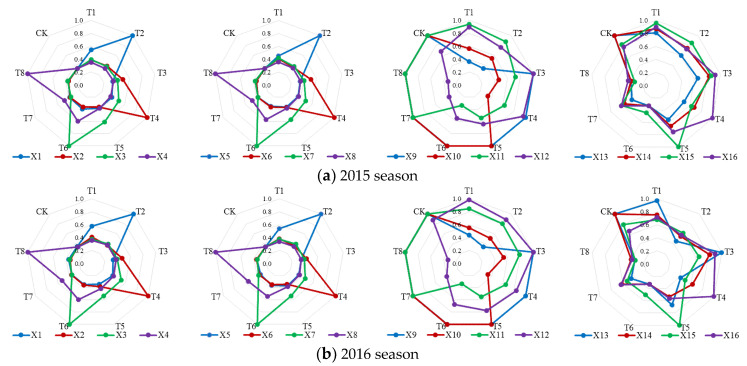
Grey relation coefficient between each index (*X*_1_–*X*_16_) and the corresponding index value in the reference sequence for nine soybean irrigation schemes (T1–T8, CK) in (**a**) 2015 and (**b**) 2016.

The absolute difference between each index and the corresponding index value in the reference sequence for each alternative scheme was calculated by Equation (8). According to the results of absolute difference, the minimum and maximum values were, respectively, 0 and 1. Then, substituting the absolute difference into Equation (9), the corresponding grey relation coefficients of sixteen indices (*X*_1_–*X*_16_) for schemes T1–T8 and CK were obtained (Figure 4). Furthermore, irrigation scheme decision-making was conducted from the perspective of a single index.

In a crop water consumption subsystem (*X*_1_–*X*_8_), for index *X*_1_, the grey relation coefficients in alternative irrigation scheme T2 were the largest (1.000) during the two seasons. The coefficient in T1 (0.542 in 2015 and 0.574 in 2016) was only lower than that in T2. Moreover, the coefficient results of *X*_5_, which reflected the irrigation amount at the seedling stage, were consistent with those of *X*_1_. Similar findings were found in evapotranspiration and irrigation amount indices at the other three stages. Therefore, soybean water consumption under water deficit was less than that under full irrigation condition at each growth stage, and the more severe the deficit, the greater the decrease of evapotranspiration. Our results were consistent with Chen et al. [35] and Li et al. [42], who studied tomato and rice evapotranspiration under deficit irrigation conditions in solar greenhouse and lysimeter plot experiments, respectively.

In a crop growth process subsystem (*X*_9_–*X*_15_), the maximum and minimum grey relation coefficients of *X*_14_ were, respectively, in CK (1.000) and T6 (0.333), for both seasons. It indicated that from the aspect of seed yield, the optimal and worst schemes were those with full irrigation during the whole growth period and serious-deficit irrigation at the flowering-podding stage, respectively. Water deficit negatively influenced soybean seed formation, especially the deficit during the reproductive growth phase. A similar result was presented by Foroud et al. [43] in a field research. In addition, according to the coefficient results of *X*_15_, the optimal and worst alternative schemes were, respectively, T5 and T8. It reflected that slight drought stress at the flowering-podding stage did not significantly decrease the number of pods, and, meanwhile, re-watering during the following period guaranteed the filling of single seed. However, severe water deficit at the seed filling stage seriously impeded the seed expansion. Our finding was consistent with that of Desclaux et al. [34] in a pot experiment, who found that drought stress at all soybean growth stages would not induce an obvious weight reduction of single seed, except for the seed filling stage.

For *X*_16_, the grey relation coefficients in T4 (1.000), T3 (0.925 in 2015 and 0.898 in 2016), and T1 (0.899 in 2015 and 0.715 in 2016) were relatively larger during the two seasons. Therefore, moderate-deficit irrigation at the vegetative growth period promoted soybean water use efficiency. In addition, severe-deficit irrigation at the seedling stage (T2) significantly decreased the seed yield (*X*_14_) and water use efficiency (*X*_16_). Similarly, Foroud et al. [43] discovered that drought stress at the soybean vegetative phase did not markedly influence yield components by a field experiment.

The decision-making results from a single index aspect could not completely consider all the information, and these alternative schemes should be comprehensively evaluated and sorted by combining the grey relation coefficient of each index with the corresponding index weight.

### 3.3. Grey Entropy Weight of Each Decision-Making Index

#### 3.3.1. Weight of Each Decision-Making Subsystem

Experts were invited to compare the importance of three subsystems in Table 6 in pairs, and the following fuzzy complementary judgment matrix *A*_sub_ was obtained. Then, substituting *A*_sub_ into Equation (11) and applying the AGA method to solve the optimization problem, where *d* was 0.2 [28], the corrected matrix *B*_sub_ and the weights of three subsystems were calculated (Table 8).

$$A_{\mathrm{sub}}=\left[\begin{array}{ccc} 0.50 & 0.55 & 0.45\\ 0.45 & 0.50 & 0.40\\ 0.55 & 0.60 & 0.50 \end{array}\right],\;\;\;B_{\mathrm{sub}}=\left[\begin{array}{ccc} 0.50 & 0.55 & 0.45\\ 0.45 & 0.50 & 0.40\\ 0.55 & 0.60 & 0.50 \end{array}\right].$$

The *CIC* of *A*_sub_ (0.000 in Table 8) was lower than 0.20, indicating that *A*_sub_ had a satisfactory consistency, and the obtained subsystem weights were acceptable. The weight of the crop water use efficiency subsystem (0.383 in Table 8) was the largest. It reflected that an optimal scheme was mainly determined by the balance between water consumption and crop production. This was in accordance with the primary targets of irrigation scheme decision-making.

#### 3.3.2. Weight of Each Decision-Making Index in the Respective Subsystem

**Figure 5:**
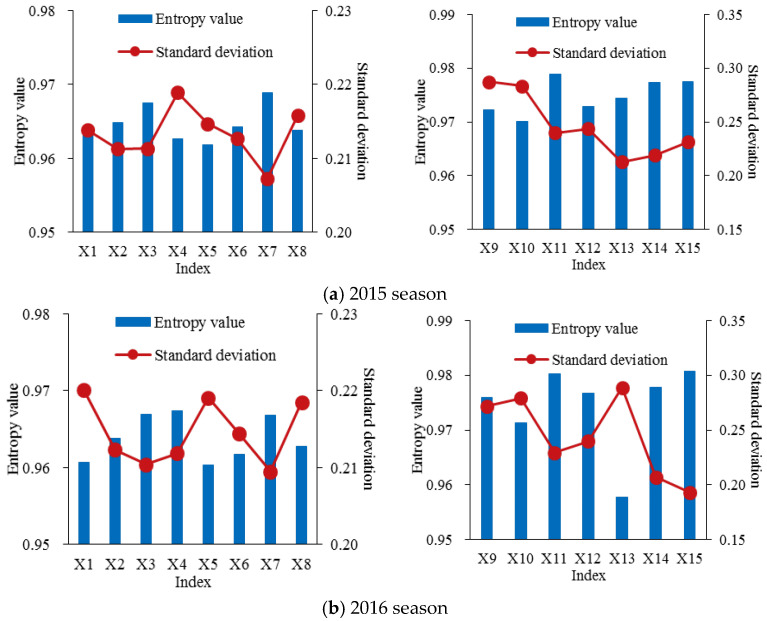
Entropy value and standard deviation of grey relation coefficient series for each soybean irrigation scheme decision-making index in (**a**) 2015 and (**b**) 2016.

Substituting the grey relation coefficient of each index for nine alternative irrigation schemes into Equations (12) and (13) in sequence, the entropy values of grey relation coefficient series for sixteen indices were obtained and are shown in Figure 5. In addition, the standard deviation of the coefficient series was calculated to quantify the dispersion degree as shown in Figure 5.

In a crop water consumption subsystem, the entropy value of grey relation coefficient series for index *X*_7_ was the largest during the 2015 season (0.969). According to information entropy theory, it indicated that the dispersion degree of this series was the lowest and meanwhile, the decision-making information provided by this series was minimal. Therefore, the grey entropy weight of *X*_7_ should be the smallest. Moreover, the standard deviation of grey relation coefficient series for *X*_7_ was the lowest (0.207) in 2015. Similarly, the entropy values for *X*_4_ and *X*_5_ were relatively small (0.963 and 0.962), while the corresponding standard deviations were relatively high (0.219 and 0.215). However, during the 2016 season, there were a large entropy value (0.967) and a small standard deviation (0.212) for *X*_4_. Therefore, the dispersion degree of grey relation coefficient series for this index is low and the corresponding grey entropy weight should be small.

In a crop growth process subsystem, the entropy values for *X*_11_ (0.979 in 2015 and 0.980 in 2016) were both the maximum during the two seasons. Those for *X*_10_ (0.970) and *X*_13_ (0.958) were the minimum in 2015 and 2016, respectively. Moreover, the results of standard deviations for these indices reflected the same dispersion degree as the entropy values in both seasons (Figure 5).

On the whole, the variations in the entropy value of grey relation coefficient series for each index in the respective subsystem during the two seasons were basically consistent (Figure 5). Furthermore, the results of entropy value accorded with those of standard deviation. Therefore, the obtained grey relation entropy value for each decision-making index was reasonable and reliable.

The previous studies [22,23,24,25,26,28,38] mostly used the original index value to calculate the corresponding entropy value when applying the entropy weight method. However, it is not reasonable to always utilize the information provided by the original index for different study objects. An object-oriented entropy weight method should, therefore, be considered. For instance, when evaluating the comprehensive grade of all courses for each student in a class, the information provided by the series of the original course scores was effective, and the series should be used to calculate the corresponding entropy value and the weight of each course. Nevertheless, when assessing the pass rate of all courses for each student, the information provided by the series of the original course scores may be redundant. Specifically, whether the course score was higher than a critical value of passing should be judged first and then the series consisted of 1 (not pass) and 2 (pass) was obtained. Accordingly, for the study object of pass rate, the information provided by this series was more direct, and this series was more effective to determine the weight of each course.

Substituting the entropy values of grey relation coefficient for each index into Equation (14), $A_{e,2015}^{1}$, $A_{e,2015}^{2}$, $A_{e,2016}^{1}$ and $A_{e,2016}^{2}$ were obtained. Then, these matrices were substituted into Equation (15), and the weight of each index in the respective subsystem was determined based on the AGA-FAHP method (Table 8), where *d* was 0.2 [28]. Accordingly, the corresponding optimal consistency judgment matrices $B_{e,2015}^{1}$, $B_{e,2015}^{2}$, $B_{e,2016}^{1}$ and $B_{e,2016}^{2}$ were calculated:

$$A_{e,2015}^{1}=\left[\begin{array}{cccccccc} 0.50 & 0.51 & 0.53 & 0.49 & 0.48 & 0.50 & 0.54 & 0.50\\ 0.49 & 0.50 & 0.52 & 0.48 & 0.48 & 0.50 & 0.53 & 0.49\\ 0.47 & 0.48 & 0.50 & 0.47 & 0.46 & 0.48 & 0.51 & 0.47\\ 0.51 & 0.52 & 0.53 & 0.50 & 0.49 & 0.51 & 0.55 & 0.51\\ 0.52 & 0.52 & 0.54 & 0.51 & 0.50 & 0.52 & 0.55 & 0.51\\ 0.50 & 0.50 & 0.52 & 0.49 & 0.48 & 0.50 & 0.53 & 0.50\\ 0.46 & 0.47 & 0.49 & 0.45 & 0.45 & 0.47 & 0.50 & 0.46\\ 0.50 & 0.51 & 0.53 & 0.49 & 0.49 & 0.50 & 0.54 & 0.50 \end{array}\right],B_{e,2015}^{1}=\left[\begin{array}{cccccccc} 0.50 & 0.51 & 0.53 & 0.48 & 0.50 & 0.50 & 0.54 & 0.49\\ 0.49 & 0.50 & 0.51 & 0.48 & 0.48 & 0.49 & 0.52 & 0.49\\ 0.47 & 0.49 & 0.50 & 0.46 & 0.47 & 0.48 & 0.51 & 0.48\\ 0.52 & 0.52 & 0.54 & 0.50 & 0.51 & 0.52 & 0.55 & 0.51\\ 0.50 & 0.52 & 0.53 & 0.49 & 0.50 & 0.52 & 0.55 & 0.51\\ 0.50 & 0.51 & 0.52 & 0.48 & 0.48 & 0.50 & 0.54 & 0.50\\ 0.46 & 0.48 & 0.49 & 0.45 & 0.45 & 0.46 & 0.50 & 0.46\\ 0.51 & 0.51 & 0.52 & 0.49 & 0.49 & 0.50 & 0.54 & 0.50 \end{array}\right]$$

$$A_{e,2015}^{2}=\left[\begin{array}{ccccccc} 0.50 & 0.48 & 0.57 & 0.51 & 0.52 & 0.55 & 0.55\\ 0.52 & 0.50 & 0.59 & 0.52 & 0.54 & 0.57 & 0.57\\ 0.43 & 0.41 & 0.50 & 0.44 & 0.45 & 0.48 & 0.49\\ 0.49 & 0.48 & 0.56 & 0.50 & 0.51 & 0.54 & 0.55\\ 0.48 & 0.46 & 0.55 & 0.49 & 0.50 & 0.53 & 0.53\\ 0.45 & 0.43 & 0.52 & 0.46 & 0.47 & 0.50 & 0.50\\ 0.45 & 0.43 & 0.51 & 0.45 & 0.47 & 0.50 & 0.50 \end{array}\right],B_{e,2015}^{2}=\left[\begin{array}{ccccccc} 0.50 & 0.48 & 0.56 & 0.51 & 0.52 & 0.55 & 0.55\\ 0.52 & 0.50 & 0.58 & 0.52 & 0.54 & 0.57 & 0.56\\ 0.44 & 0.42 & 0.50 & 0.44 & 0.46 & 0.48 & 0.48\\ 0.49 & 0.48 & 0.56 & 0.50 & 0.51 & 0.54 & 0.54\\ 0.48 & 0.46 & 0.54 & 0.49 & 0.50 & 0.54 & 0.53\\ 0.45 & 0.43 & 0.52 & 0.46 & 0.46 & 0.50 & 0.50\\ 0.45 & 0.44 & 0.52 & 0.46 & 0.47 & 0.50 & 0.50 \end{array}\right]$$

$$A_{e,2016}^{1}=\left[\begin{array}{cccccccc} 0.50 & 0.52 & 0.54 & 0.55 & 0.50 & 0.51 & 0.54 & 0.51\\ 0.48 & 0.50 & 0.52 & 0.53 & 0.48 & 0.49 & 0.52 & 0.49\\ 0.46 & 0.48 & 0.50 & 0.50 & 0.45 & 0.46 & 0.50 & 0.47\\ 0.45 & 0.47 & 0.50 & 0.50 & 0.45 & 0.46 & 0.50 & 0.47\\ 0.50 & 0.52 & 0.55 & 0.55 & 0.50 & 0.51 & 0.54 & 0.52\\ 0.49 & 0.51 & 0.54 & 0.54 & 0.49 & 0.50 & 0.54 & 0.51\\ 0.46 & 0.48 & 0.50 & 0.50 & 0.46 & 0.46 & 0.50 & 0.47\\ 0.49 & 0.51 & 0.53 & 0.53 & 0.48 & 0.49 & 0.53 & 0.50 \end{array}\right],B_{e,2016}^{1}=\left[\begin{array}{cccccccc} 0.50 & 0.52 & 0.55 & 0.54 & 0.50 & 0.50 & 0.54 & 0.51\\ 0.48 & 0.50 & 0.53 & 0.52 & 0.48 & 0.48 & 0.53 & 0.49\\ 0.45 & 0.47 & 0.50 & 0.50 & 0.46 & 0.47 & 0.50 & 0.47\\ 0.46 & 0.48 & 0.50 & 0.50 & 0.46 & 0.47 & 0.50 & 0.48\\ 0.50 & 0.52 & 0.54 & 0.54 & 0.50 & 0.51 & 0.55 & 0.52\\ 0.50 & 0.52 & 0.53 & 0.53 & 0.49 & 0.50 & 0.54 & 0.50\\ 0.46 & 0.47 & 0.50 & 0.50 & 0.45 & 0.46 & 0.50 & 0.48\\ 0.49 & 0.51 & 0.53 & 0.52 & 0.48 & 0.50 & 0.52 & 0.50 \end{array}\right]$$

$$A_{e,2016}^{2}=\left[\begin{array}{ccccccc} 0.50 & 0.46 & 0.55 & 0.51 & 0.36 & 0.52 & 0.56\\ 0.54 & 0.50 & 0.59 & 0.55 & 0.40 & 0.56 & 0.60\\ 0.45 & 0.41 & 0.50 & 0.46 & 0.32 & 0.47 & 0.51\\ 0.49 & 0.45 & 0.54 & 0.50 & 0.36 & 0.51 & 0.55\\ 0.64 & 0.60 & 0.68 & 0.64 & 0.50 & 0.66 & 0.69\\ 0.48 & 0.44 & 0.53 & 0.49 & 0.34 & 0.50 & 0.54\\ 0.44 & 0.40 & 0.49 & 0.45 & 0.31 & 0.46 & 0.50 \end{array}\right],B_{e,2016}^{2}=\left[\begin{array}{ccccccc} 0.50 & 0.46 & 0.55 & 0.50 & 0.37 & 0.53 & 0.56\\ 0.54 & 0.50 & 0.59 & 0.53 & 0.41 & 0.56 & 0.59\\ 0.45 & 0.41 & 0.50 & 0.45 & 0.33 & 0.47 & 0.51\\ 0.50 & 0.47 & 0.55 & 0.50 & 0.36 & 0.51 & 0.55\\ 0.63 & 0.59 & 0.67 & 0.64 & 0.50 & 0.66 & 0.69\\ 0.47 & 0.44 & 0.53 & 0.49 & 0.34 & 0.50 & 0.54\\ 0.44 & 0.41 & 0.49 & 0.45 & 0.31 & 0.46 & 0.50 \end{array}\right].$$

The *CIC* of the matrices for the crop water consumption subsystem (0.004 and 0.004) and crop growth process subsystem (0.003 and 0.004) in 2015 and 2016 were all less than 0.20 (Table 8). Therefore, the obtained grey entropy weights of all indices in the two subsystems were acceptable.

In the crop water consumption subsystem for 2015, the grey entropy weights of indices *X*_4_ (0.130) and *X*_5_ (0.129) were relatively large, and that of *X*_7_ (0.116) was the smallest. Similarly, the weights of *X*_5_ (0.131) and *X*_7_ (0.118) were, respectively, the maximum and minimum in 2016. These results were in accordance with the fact that the entropy values of grey relation coefficient series for *X*_5_ and *X*_7_ were, respectively, relatively low and high in Figure 5. Moreover, in the crop growth process subsystem for 2015, the weights of *X*_10_ (0.157) and *X*_11_ (0.130) were the highest and lowest, respectively. However, the weight of *X*_13_ (0.185) was the maximum and that of *X*_11_ (0.125) was relatively small during the 2016 season (Table 8).

The grey entropy weights of each index in the respective subsystem for the two seasons were basically consistent. Furthermore, the grey entropy weight results were in accordance with those of the entropy values of grey relation coefficient series for each index in Figure 5. Therefore, the grey entropy weight method according to the information provided by the grey relation coefficient series of each decision-making index was reasonable, and the obtained index weights were reliable.

The grey entropy weight proposed in this study could be regarded as one object-oriented entropy weight method, and it could be implemented to explain interdecadal variations based on long time series [44]. Therefore, the present research with samples of two cropping seasons would be further explored and analyzed according to samples of monthly time series in our further work.

The comprehensive grey entropy weight of each decision-making index was calculated according to Equation (16). The comprehensive weights of fifteen indices (*X*_1_–*X*_15_) distributed evenly, and those of *X*_13_ and *X*_11_ were both relatively large and small in the two seasons (Table 8).

### 3.4. Grey Relation Projection Weight of Each Decision-Making Index

**Figure 6:**
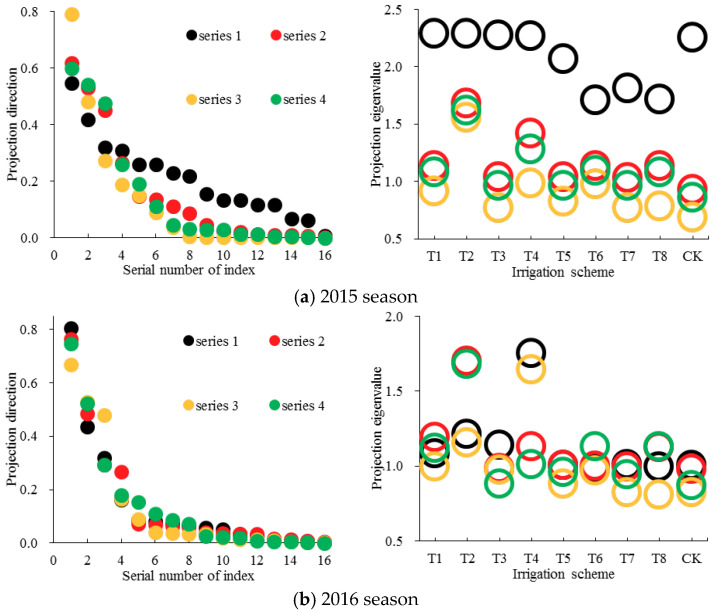
Projection directions of each decision-making index and the corresponding one-dimensional projection eigenvalue of each soybean irrigation scheme for the first four optimized results during the (**a**) 2015 and (**b**) 2016 seasons.

The initial projection directions (random number from 0 to 1) and grey relation coefficients of sixteen indices for nine alternative irrigation schemes were substituted into Equation (17), respectively, and the one-dimensional projection eigenvalue for each scheme was obtained. Then, according to Equations (18)–(20), the projection index function was constructed. Using the AGA method to solve the optimization issue in Equation (21), the optimized projection vector was calculated. The projection direction of each decision-making index (sorted from large to small according to the projection direction values) and the corresponding projection eigenvalue of each alternative scheme for the first four optimization results (series 1 to series 4) are shown in Figure 6.

During the 2015 season, the projection direction values of most indices were close to 0 in series 2, series 3 and series 4. However, those of sixteen indices in series 1 were distributed more evenly. Accordingly, the projection eigenvalues of nine schemes in series 1 satisfied the required distribution characteristic more precisely (Figure 6). Specifically, the values of *S*_Z_ in series 1 to series 4 were, respectively, 0.258, 0.234, 0.260 and 0.229, the values of *D*_Z_ were, respectively, 0.435, 0.456, 0.378 and 0.406. Therefore, the local projection points in series 1 were more concentrated and, meanwhile, the overall distribution of all projection points was more scattered. The results of projection direction for each decision-making index based on series 1 (the corresponding maximum value of objective function *Q* in Equation (21) was 0.111) were adopted (Table 9).

In 2016, the differences among the projection directions of sixteen indices and the projection eigenvalues of nine alternative schemes were both not significant for the four series (Figure 6). Moreover, the values of *S*_Z_*D*_Z_ in series 1 to series 4 were 0.111, 0.107, 0.103 and 0.097, respectively. Therefore, the optimized projection direction value of each decision-making index in series 1 (the corresponding maximum value of objective function *Q* in Equation (21) was 0.112) were further used to calculate the corresponding grey relation projection weight (Table 9).

Substituting the optimal projection direction value of each index (Table 9) into Equation (22), the grey relation projection weights of sixteen indices were obtained (Table 8). The weights of indices *X*_12_ (0.299) and *X*_6_ (0.001) were, respectively, the maximum and minimum in 2015. However, those of *X*_2_ (0.644) and *X*_10_ (0.001) were the highest and lowest, respectively, in 2016. The difference in index weight for the two seasons may be caused by the difference in the distance of grey relation coefficients between every two schemes. Moreover, the combined weights of sixteen indices were calculated (Table 8) according to Equation (24), and the corresponding minimum values of objective function *F* in Equation (23) were 0.596 and 1.425 during the 2015 and 2016 seasons, respectively.

### 3.5. Decision-Making Results of Irrigation Scheme for Soybeans in the Huaibei Plain

**Figure 7:**
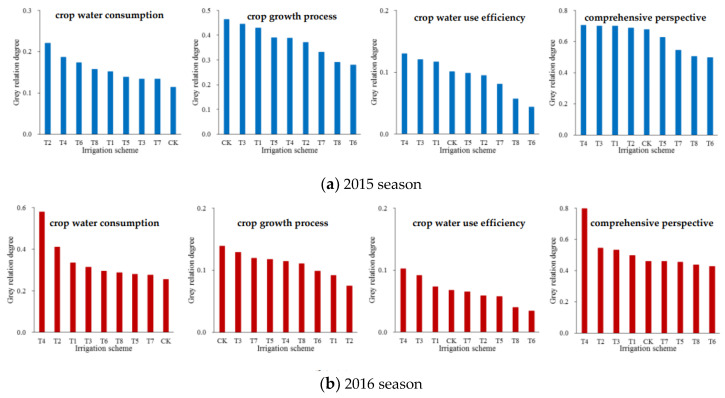
Grey relation degree between each alternative irrigation scheme and the ideal scheme for soybean in the Huaibei Plain from different perspectives during the (**a**) 2015 and (**b**) 2016 seasons.

By multiplying the grey relation coefficient of each index by the corresponding combined index weight and calculating the sum of these products for all indices according to Equation (25), the grey relation degree between each alternative scheme and the ideal scheme was obtained and is shown in Figure 7. Furthermore, the alternative schemes were sorted based on the degree values, and the larger the degree value of an alternative scheme was, the more effective the scheme was.

In a crop water consumption subsystem, the values of grey relation degree in T2 (0.220 and 0.411) and T4 (0.187 and 0.580) were relatively high, and those in CK (0.114 and 0.254) and T7 (0.134 and 0.276) were relatively low during the 2015 and 2016 seasons, respectively. It indicated that the scheme with severe-deficit irrigation at the seedling stage or branching stage was relatively optimal from the aspect of water conservation. This was due to the fact that serious drought stress at these two stages not only decreased the soybean evapotranspiration at the current stage, but also reduced the evapotranspiration during the following periods relative to full irrigation. A similar result was obtained by Cui et al. [45], who analyzed the variations of winter wheat evapotranspiration under drought stress conditions during several growth stages by lysimeter plot experiments.

In a crop growth process subsystem, the degree values in CK (0.463 and 0.139) and T3 (0.445 and 0.129) were both relatively large for 2015 and 2016. It reflected that the scheme with full irrigation during the whole growth period and that with slight-deficit irrigation at the branching stage guaranteed normal soybean growth and seed formation. This was directly related to the relatively sufficient evapotranspiration achieved under these two abundant water supply conditions. Similarly, Kendig et al. [46] found that soybean yields were the highest under full-season irrigation treatment, followed by irrigation initiated at flowering, irrigation terminated at flowering and no irrigation treatments based on field experiments in Fayetteville.

In a crop water use efficiency subsystem, the degree values for nine alternative schemes were basically consistent during the two seasons. Deficit irrigation at the branching stage (T3 and T4) improved soybean water use efficiency. Moreover, serious drought stress during the reproductive growth phase did not effectively reduce the soybean water consumption, but severely obstructed the seed formation and greatly decreased the water use efficiency (Figure 7). This was in agreement with the findings of Dogan et al. [13] and Foroud et al. [43] by field experiments. In addition, Lopez et al. [47] found that the water use efficiency with irrigation only during soybean reproductive stage R3 was 18% higher than that under the well-watered condition in Gainesville by model simulation.

According to the values of comprehensive grey relation degree from large to small, the optimal alternative scheme was in the order of T4, T3, T1, T2, CK, T5, T7, T8 and T6 during the 2015 season (Figure 7). Similarly, the sequence was T4, T2, T3, T1, CK, T7, T5, T8 and T6 in 2016 (Figure 7). Therefore, from an integrated perspective of water conservation, large production and high efficiency, the optimal scheme was that with severe-deficit irrigation at the branching stage (0.704 in 2015 and 0.797 in 2016), and the superiority of this irrigation scheme was significant (Figure 7). The worst scheme was that with serious-deficit irrigation at the flowering-podding stage (0.496 in 2015 and 0.428 in 2016). In addition, the schemes with deficit irrigation during the soybean vegetative growth phase (the seedling and branching stages) were more effective than those during the reproductive growth phase (the flowering-podding and seed filling stages), and full irrigation during the whole growth period was a moderate scheme. Similarly, Zhang et al. [48] presented that guaranteeing the soil water content at the seedling and branching stages required no less than 70% of field capacity, and that at the flowering-podding and seed filling stages, no less than 80% of field capacity provided a suitable regulated irrigation scheme for soybean in Northeast China as determined by an irrigation scheme decision-making study with a pot experiment.

In addition, the scheme decision-making results in this study were consistent with some studies on soybean irrigation schedule in China. Li et al. [18] recommended that soybeans should be irrigated three times during the growing season (at sowing, the middle floral differentiation stage and the middle seed-setting stage, respectively) and each irrigation amount was 40 mm in western Jilin Province from a perspective of crop water requirement by experiments. Fu et al. [49] proposed that the useful irrigation quota of a normal year for soybean in the downstream Songhua River Basin was 28 mm, with six times that during the pod formation and seed enlargement stages based on the SWAT (Soil and Water Assessment Tool) model. Moreover, Zhang et al. [31] presented that soybeans in the Huaibei District of Anhui Province should be irrigated one time (at the pod-filling stage) and two times (both at the flowering and podding stage) for the normal and dry years, respectively, and the irrigation amount per time was 45 mm as determined by an optimization model and years of experiments. In a word, the optimal scheme obtained in our study provides effective guidance for formulating an accurate irrigation schedule consisting of irrigation times and irrigation amount per time in the Huaibei Plain. However, years of temporal distributions for precipitation and groundwater during the whole soybean growth period and field experiments with various levels of deficit irrigation at the seedling and branching stages should be further implemented and analyzed.

Deficit irrigation at the soybean vegetative growth phase not only decreases the water consumption but also ensures the biomass yield. Specifically, the influence of water deficit during this period could be further transmitted, which results in a reduction of evapotranspiration at the following growth stages relative to sufficient irrigation [45]. Furthermore, soybean plants may be able to recover from the deficit influence and return to normal growth after re-watering at the next stages [45]. Similarly, Desclaux et al. [34] discovered that early drought stress during the soybean vegetative period primarily decreased the biomass and internode length in a pot experiment. In addition, soybeans may have a certain tolerance to water deficit after normal growth during the seedling stage, which causes a more effective scheme with deficit irrigation at the branching stage.

Deficit irrigation at the reproductive phase greatly impedes the seed formation. Thus, slight-deficit irrigation during this period is relatively optimal compared to serious-deficit irrigation. At the flowering and pod-enlargement stage, vegetative and reproductive growth both proceed and water consumption approaches the maximum [50]. Once soybean plants encounter a serious water deficit at this period, the pod expansion and seed formation would be significantly affected [51,52].

In conclusion, the comprehensive decision-making results from the perspective of soybean growth responses at each stage for different irrigation schemes were mostly consistent in 2015 and 2016. The scheme with moderate-deficit irrigation at the branching stage or seedling stage and adequate irrigation at the flowering-podding and seed filling stages was relatively optimal for an integrated target of lower water consumption and stable biomass yields, which provided an effective reference for formulating an accurate soybean irrigation schedule in the Huaibei Plain.

## 4. Conclusions

In this paper, an irrigation scheme decision-making index system was constructed from the perspectives of crop water consumption, crop growth process and crop water use efficiency. Moreover, a grey entropy weight method and a grey relation–projection pursuit model were proposed to calculate the weight of each decision-making index. Then, nine alternative irrigation schemes based on soybean water deficit experiments during two cropping seasons in the Huaibei Plain were sorted according to the comprehensive grey relation degree of each scheme.

When using the entropy weight method or projection pursuit model to calculate index weight, it was more effective to calculate the corresponding entropy value or projection eigenvalue according to the sequence of the actual study object, rather than that of the original index value.

The scheme with severe-deficit irrigation at the seedling stage or branching stage was relatively optimal from the aspect of water conservation. Serious water deficit during these two periods not only decreased the soybean evapotranspiration at the current stage but also reduced the evapotranspiration during the following stages relative to full irrigation condition.

The scheme with full irrigation during the whole growth period and that with slight-deficit irrigation at the branching stage guaranteed normal soybean growth and seed formation. This was related to the sufficient evapotranspiration achieved under these two abundant water conditions.

Deficit irrigation at the branching stage improved soybean water use efficiency. Moreover, serious drought stress during the reproductive growth phase did not effectively reduce water consumption, but severely obstructed seed formation and greatly decreased water use efficiency.

The comprehensive decision-making results from the perspective of soybean growth responses at each stage for different irrigation schemes were mostly consistent in the 2015 and 2016 seasons. The scheme with moderate-deficit irrigation at the soybean branching stage or seedling stage and adequate irrigation at the flowering-podding and seed filling stages was relatively optimal for an integrated target of lower water consumption and stable biomass yields in the Huaibei Plain.

Grey entropy weight can be considered as one object-oriented entropy weight method, which can be further applied according to different study objects. In addition, the optimal scheme results obtained in this study provide an effective reference for determining an accurate soybean irrigation schedule in the Huaibei Plain. Furthermore, years of temporal distributions for precipitation and groundwater during the whole soybean growth period and field experiments with various levels of deficit irrigation at the seedling and branching stages would be conducted in our future works.

## Figures and Tables

**Table 1:** Characteristics of the experimental soil at the upper layer (0–50 cm).

Soil Characteristics	Value
Sand (%)	3.45
Silt (%)	70.52
Clay (%)	26.03
pH (in water solution)	7.5
Organic matter (%)	0.85
Bulk density (g/cm^3^)	1.36
Field capacity at −0.03 MPa (cm^3^/cm^3^)	0.38
Wilting point at −1.5 MPa (cm^3^/cm^3^)	0.12

**Table 2:** Cultivar parameters of the soybean seed (cv. Zhonghuang-13) in the experiments.

Cultivar Parameters	Value	Seed Characteristics		Value
Whole growth period (d)	97	Protein content (%)		43.73
Plant height (cm)	46.3	Oil content (%)		19.10
Number of nods on main stem	13.8	Vitamin E content (μg/g)		181.9 ± 25.1
Number of branches per plant	2.3	Fatty acid (%)	16: 0	11.5 ± 0.5
Number of pods per plant	40.0		18: 0	4.2 ± 0.4
Number of seeds per pod	2.04		18: 1	25.5 ± 0.5
Weight of 100 seeds (g)	24.0		18: 2	51.8 ± 0.8
Seed yield (t/ha)	3.04		18: 3	7.0 ± 0.2

**Table 3:** Divisions of the whole soybean growth periods in the 2015 and 2016 seasons.

Description of Growth Stage	2015 Season	2016 Season
Germination stage, from sowing to seed germination	From June 20 to July 3, 14 days	From June 29 to July 14, 16 days
Seedling stage, from seed germination to plants with four fully expanded leaves	From July 4 to July 14, 11 days	From July 15 to July 27, 13 days
Branching stage, from plants with four fully expanded leaves to first flower appearance	From July 15 to August 3, 20 days	From July 28 to August 10, 14 days
Flowering-podding stage, from first flower appearance to the beginning of pod filling	From August 4 to August 20, 17 days	From August 11 to August 31, 21 days
Seed filling stage, from the beginning of pod filling to plant maturation	From August 21 to September 20, 31 days	From September 1 to September 27, 27 days

**Table 4:** Average values of daily meteorological elements during each soybean growth stage in the 2015 and 2016 seasons.

Meteorological Element	Germination Stage		Seedling Stage		Branching Stage		Flowering-Podding Stage		Seed Filling Stage	
	2015	2016	2015	2016	2015	2016	2015	2016	2015	2016
Maximum air temperature (°C)	27.8	29.8	30.9	33.8	32.2	34.2	31.5	32.8	28.9	30.6
Minimum air temperature (°C)	20.9	23.3	21.3	24.7	24.1	25.7	22.8	22.7	17.6	18.4
Mean air temperature (°C)	24.0	26.3	26.0	29.1	28.0	29.3	26.7	27.7	22.9	24.2
Mean air relative humidity (%)	88.4	87.8	82.2	81.3	85.9	84.2	86.4	79.3	86.7	74.8
Wind speed (m/s)	0.9	1.3	0.9	1.0	1.0	1.0	0.9	1.0	0.6	0.9
Sunshine duration (h)	0.5	3.5	2.8	6.4	3.6	7.4	5.3	7.4	7.3	6.6
Solar radiation (MJ/(m^2^·d))	9.92	12.30	14.37	18.03	13.32	16.48	13.48	16.73	15.12	13.95
Vapor pressure deficit (kPa)	0.36	0.42	0.63	0.80	0.58	0.69	0.50	0.75	0.37	0.76
Reference evapotranspiration (mm/d)	2.33	3.23	3.09	4.28	3.42	4.43	3.56	4.11	3.29	3.18

**Table 5:** Percentage of lower limits of soil water content relative to field capacity at each soybean growth stage for different irrigation schemes in the 2015 and 2016 seasons.

Cropping Season	Irrigation Scheme	Seedling Stage	Branching Stage	Flowering-Podding Stage	Seed Filling Stage
2015 and 2016	T1	55%	75%	75%	75%
	T2	35%	75%	75%	75%
	T3	75%	55%	75%	75%
	T4	75%	35%	75%	75%
	T5	75%	75%	55%	75%
	T6	75%	75%	35%	75%
	T7	75%	75%	75%	55%
	T8	75%	75%	75%	35%
	CK	75%	75%	75%	75%

**Table 6:** Irrigation scheme decision-making index system for soybeans in the Huaibei Plain.

Decision-Making System	Decision-Making Index	Index Type
crop water consumption subsystem	***X*_1_** soybean evapotranspiration at the seedling stage (mm)	negative
	***X*_2_** soybean evapotranspiration at the branching stage (mm)	negative
	***X*_3_** soybean evapotranspiration at the flowering-podding stage (mm)	negative
	***X*_4_** soybean evapotranspiration at the seed filling stage (mm)	negative
	***X*_5_** irrigation amount at the seedling stage (mm)	negative
	***X*_6_** irrigation amount at the branching stage (mm)	negative
	***X*_7_** irrigation amount at the flowering-podding stage (mm)	negative
	***X*_8_** irrigation amount at the seed filling stage (mm)	negative
crop growth process subsystem	***X*_9_** soybean aboveground accumulated biomass at the seedling stage (t/ha)	positive
	***X*_10_** soybean aboveground accumulated biomass at the branching stage (t/ha)	positive
	***X*_11_** soybean aboveground accumulated biomass at the flowering-podding stage (t/ha)	positive
	***X*_12_** soybean aboveground accumulated biomass at the seed filling stage (t/ha)	positive
	***X*_13_** soybean aboveground biomass at harvest time (t/ha)	positive
	***X*_14_** soybean seed yield (t/ha)	positive
	***X*_15_** soybean 1000 seed weight (g)	positive
crop water use efficiency subsystem	***X*_16_** soybean water use efficiency during the whole growth period (kg/m^3^)	positive

**Table 7:** Normalized value of each index for soybean irrigation scheme decision-making.

Cropping Season	Irrigation Scheme	Crop Water Consumption Decision-Making Subsystem								Crop Growth Process Decision-Making Subsystem							Crop Water Use Efficiency Decision-Making Subsystem
		*X*_1_	*X*_2_	*X*_3_	*X*_4_	*X*_5_	*X*_6_	*X*_7_	*X*_8_	*X*_9_	*X*_10_	*X*_11_	*X*_12_	*X*_13_	*X*_14_	*X*_15_	*X*_16_
2015	T1	0.58	0.21	0.23	0.07	0.39	0.26	0.31	0.09	0.13	0.62	0.97	0.94	0.88	0.93	0.98	0.94
	T2	1.00	0.21	0.16	0.02	1.00	0.11	0.17	0.05	0.00	0.58	0.93	0.84	0.67	0.83	0.92	0.81
	T3	0.01	0.50	0.30	0.07	0.03	0.53	0.27	0.07	1.00	0.42	0.81	1.00	0.74	0.89	0.92	0.96
	T4	0.16	1.00	0.48	0.07	0.09	1.00	0.48	0.10	1.00	0.00	0.70	0.98	0.49	0.76	0.71	1.00
	T5	0.18	0.11	0.67	0.19	0.11	0.13	0.62	0.19	1.00	1.00	0.58	0.72	0.60	0.75	1.00	0.84
	T6	0.22	0.11	1.00	0.66	0.07	0.11	1.00	0.62	1.00	1.00	0.00	0.59	0.00	0.00	0.37	0.00
	T7	0.18	0.17	0.12	0.45	0.16	0.15	0.15	0.44	1.00	1.00	1.00	0.11	0.33	0.61	0.68	0.69
	T8	0.09	0.09	0.14	1.00	0.08	0.08	0.15	1.00	1.00	1.00	1.00	0.00	0.22	0.22	0.00	0.36
	CK	0.00	0.00	0.00	0.00	0.00	0.00	0.00	0.00	1.00	1.00	1.00	0.76	1.00	1.00	0.89	0.85
2016	T1	0.63	0.26	0.17	0.10	0.57	0.19	0.16	0.01	0.36	0.59	0.91	0.99	0.98	0.83	0.76	0.80
	T2	1.00	0.15	0.24	0.15	1.00	0.10	0.22	0.05	0.00	0.52	0.87	0.94	0.41	0.60	0.69	0.63
	T3	0.00	0.44	0.24	0.16	0.02	0.33	0.22	0.04	1.00	0.58	0.87	1.00	1.00	0.89	0.73	0.94
	T4	0.16	1.00	0.54	0.20	0.06	1.00	0.41	0.10	1.00	0.00	0.73	0.91	0.30	0.70	0.49	1.00
	T5	0.03	0.18	0.57	0.29	0.13	0.04	0.56	0.18	1.00	1.00	0.58	0.86	0.75	0.57	1.00	0.61
	T6	0.07	0.09	1.00	0.66	0.11	0.05	1.00	0.57	1.00	1.00	0.00	0.76	0.00	0.00	0.51	0.00
	T7	0.08	0.10	0.09	0.55	0.00	0.10	0.14	0.60	1.00	1.00	1.00	0.25	0.39	0.70	0.55	0.71
	T8	0.12	0.00	0.06	1.00	0.08	0.09	0.05	1.00	1.00	1.00	1.00	0.00	0.03	0.24	0.00	0.21
	CK	0.05	0.00	0.00	0.00	0.01	0.00	0.00	0.00	1.00	1.00	1.00	0.93	1.00	1.00	0.87	0.74

**Table 8:** Weight of each soybean irrigation scheme decision-making index.

Decision-Making System	Decision-Making Index	Improved Fuzzy Analytic Hierarchy Process Method		Grey Entropy Weight Method						Grey Relation–Projection Pursuit Model		Combined Weight	
		Subsystem Weight	CIC	Index Weight		CIC		Comprehensive Index Weight		2015	2016	2015	2016
				2015	2016	2015	2016	2015	2016				
crop water consumption subsystem	*X*_1_	0.334	0.000	0.127	0.131	0.004	0.004	0.043	0.044	0.174	0.191	0.116	0.186
	*X*_2_			0.124	0.125			0.042	0.042	0.094	0.644	0.084	0.335
	*X*_3_			0.120	0.119			0.040	0.040	0.005	0.005	0.018	0.030
	*X*_4_			0.130	0.120			0.043	0.040	0.018	0.001	0.038	0.004
	*X*_5_			0.129	0.131			0.043	0.044	0.014	0.003	0.033	0.025
	*X*_6_			0.126	0.128			0.042	0.043	0.001	0.102	0.002	0.135
	*X*_7_			0.116	0.118			0.039	0.039	0.017	0.001	0.035	0.004
	*X*_8_			0.126	0.128			0.042	0.043	0.004	0.008	0.017	0.037
crop growth process subsystem	*X*_9_	0.283		0.151	0.141	0.003	0.004	0.043	0.040	0.047	0.027	0.061	0.067
	*X*_10_			0.157	0.154			0.044	0.043	0.013	0.001	0.033	0.001
	*X*_11_			0.130	0.125			0.037	0.035	0.068	0.004	0.067	0.025
	*X*_12_			0.149	0.139			0.042	0.039	0.299	0.001	0.151	0.011
	*X*_13_			0.145	0.185			0.041	0.052	0.068	0.001	0.071	0.004
	*X*_14_			0.133	0.134			0.038	0.038	0.052	0.003	0.060	0.021
	*X*_15_			0.135	0.122			0.038	0.035	0.102	0.001	0.084	0.013
crop water use efficiency subsystem	*X*_16_	0.383		1.000	1.000	\	\	0.383	0.383	0.024	0.007	0.130	0.102

**Table 9:** Optimized projection index function values and the corresponding projection direction values of each index for soybean irrigation scheme decision-making.

Cropping Season	Optimized Projection Index Function *Q*(*y*) = *S*_z_*D*_z_			Projection Direction of Each Decision-Making Index (Projection Vector *y*)															
	*S*_z_	*D*_z_	*Q*	*X*_1_	*X*_2_	*X*_3_	*X*_4_	*X*_5_	*X*_6_	*X*_7_	*X*_8_	*X*_9_	*X*_10_	*X*_11_	*X*_12_	*X*_13_	*X*_14_	*X*_15_	*X*_16_
2015	0.26	0.44	0.11	0.42	0.31	0.07	0.13	0.12	0.01	0.13	0.06	0.22	0.12	0.26	0.55	0.26	0.23	0.32	0.16
2016	0.25	0.45	0.11	0.44	0.81	0.07	0.01	0.06	0.32	0.01	0.09	0.16	0.00	0.07	0.03	0.01	0.05	0.04	0.08
